# SHIP1 Restrains Hypoxia/Reoxygenation‐Induced Cytokine Secretion in Mouse Bone Marrow–Derived Mast Cells

**DOI:** 10.1002/eji.70274

**Published:** 2026-09-07

**Authors:** Lara Gubeljak, Jonas Pes, Eren Arik, Aaron T. Fehr, Mara Schietinger, Thomas Wilhelm, Sandro Capellman, Michael Huber, Pardes Habib

**Affiliations:** ^1^ Department of Neurology Medical Faculty RWTH Aachen University Aachen Germany; ^2^ Institute of Medical Microbiology Medical Faculty RWTH Aachen University Aachen Germany; ^3^ Institute of Biochemistry and Molecular Immunology Medical Faculty RWTH Aachen University Aachen Germany; ^4^ Department of Neurosurgery Stanford University School of Medicine Stanford California USA

**Keywords:** cytokine regulation, HIF‐1α, hypoxia‐induced activation, IL‐6, ischemia–reperfusion, mast cells, PI3K/AKT pathway, reoxygenation, SHIP1, TNF‐α

## Abstract

Mast cells (MCs) are widely distributed sentinel cells of the myeloid lineage with substantial migratory capacity. Their activation is initiated by receptor‐mediated signaling and culminates in the release of prestored mediators and cytokine secretion, processes tightly controlled by the lipid phosphatase SH2‐containing inositol 5′‐phosphatase 1 (SHIP1). Although MCs frequently reside within hypoxic microenvironments, the impact of hypoxia/reoxygenation on their activation remains elusive. Here we investigated the hypoxia tolerance of MCs and assessed whether hypoxia (≤1% O_2_) or subsequent reoxygenation (21% O_2_) induces degranulation and/or cytokine production. We further assessed the role of SHIP1 in posthypoxic MC activation. Primary mouse bone marrow‐derived MCs (BMMCs) from wildtype and *Ship1^−/−^* mice were subjected to defined periods of hypoxia and subsequent reoxygenation to meticulously analyze their viability, metabolic activity, degranulation, and cytokine secretion. BMMCs were much more resilient to hypoxia than other primary murine cell types, such as microglia, astrocytes, and fibroblasts. A 3 h hypoxic exposure induced sufficient hypoxia‐inducible factor‐1α (HIF‐1α) and its downstream effectors carbonic anhydrase IX and glucose transporter 1, with higher baseline expression in *Ship1* ^−/−^ BMMCs. Hypoxia increased secretion of IL‐6 and TNF‐α, but failed to trigger degranulation, even in *Ship1* ^−/−^ cells. Reoxygenation after 3 h of hypoxia further amplified cytokine secretion and HIF‐1α response. Our data indicate that hypoxia activates BMMCs, causing enhanced cytokine secretion and HIF‐1α pathway engagement. SHIP1 restrains hypoxia/reoxygenation–driven cytokine release, implicating PI3K/AKT signaling in this regulatory pathway. Recognizing this regulatory checkpoint may inform future efforts to modulate MC‐driven cytokine responses in ischemia–reperfusion and other hypoxic inflammatory settings.

## Graphical abstract

**(A)** BMMCs are substantially more resistant to oxygen deprivation than neurons, microglia, oligodendrocytes, astrocytes, and fibroblasts. **(B)** Role of SHIP‐1 in primary mouse bone marrow mast cells’ hypoxia response. Under hypoxia, SHIP‐1 induces cytokine release. SHIP1 restrains hypoxia/reoxygenation‐induced cytokine secretion in BMMCs via the RAS/ RAF, HIF1α, and PI3K/AKT pathways. The graphical abstract was prepared using BioRender.

**Alt text**: Summary illustration showing that BMMCs are more resistant to hypoxia than other primary murine cell types. Diagram depicts SHIP1 as a regulator of hypoxia responses, limiting cytokine secretion in BMMCs via pathways involving RAS/RAF, HIF‐1α, and PI3K/AKT signaling during hypoxia and reoxygenation. The graphical abstract was prepared using BioRender.

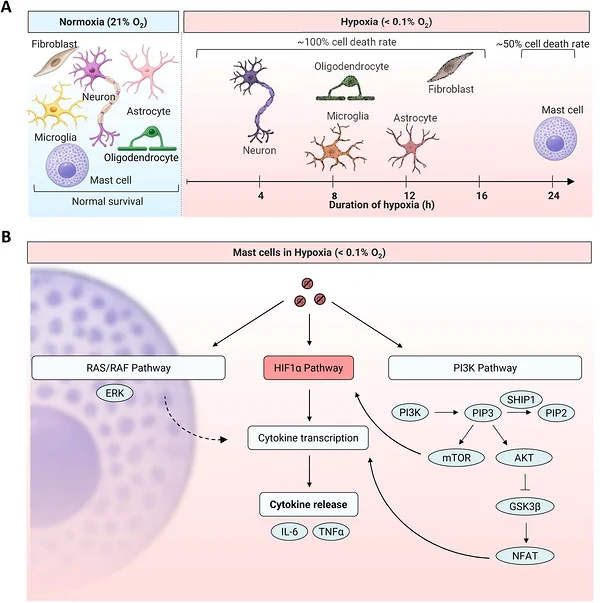

**Abbreviations**
Ag: antigen
BMMCs: bone marrow‐derived mast cells
CAIX: carbonic anhydrase IX
FBS: fetal bovine serum
FcεRI: high‐affinity IgE receptor
GLUT‐1: glucose transporter 1
HIF‐1α: hypoxia‐inducible factor 1 alpha
IL‐6: interleukin 6
IP3: inositol‐1,4,5‐trisphosphate
MCs: mast cells
MEFs: mouse embryonic fibroblasts
PBS: phosphate‐buffered saline
PIP3: phosphatidylinositol‐3,4,5‐trisphosphate
PI‐4,5‐P2: phosphatidylinositol‐4,5‐bisphosphate
pERK: phosphorylated extracellular regulated kinase
pGSK3β: phosphorylated of glycogen synthase kinase‐3β
PI3K: phosphoinositide 3‐kinase
SHIP1: SRC‐homology 2 domain‐containing inositol 5’‐phosphatase 1
TNF‐α: tumor necrosis factor alpha

## 1 Introduction

Mast cells (MCs) are tissue‐resident immune sentinels derived from myeloid progenitors that egress from the bone marrow and complete their differentiation within peripheral tissues [1]. Positioned at environmental interfaces and perivascular niches, MCs rapidly translate danger signals into local and systemic inflammation through two separable effector programs: (i) degranulation, the immediate exocytosis of preformed mediators stored in secretory lysosomes, and (ii) activation‐associated de novo mediator production, which requires enzymatic, transcriptional and translational activation, and results in the release of lipid mediators, chemokines, and cytokines [2, 3, 4]. While IgE‐loaded FcεRI crosslinking via multivalent antigen (Ag) remains the best characterized mode of MC activation, MCs integrate a broad repertoire of antigen‐dependent and antigen‐independent inputs including innate, alarmin, complement, and neuropeptide receptors. This enables context‐specific responses that can be pro‐inflammatory, immunoregulatory, or tissue‐repair oriented [5].

MCs are frequently found in hypoxic microenvironments, and their activation is involved in the pathophysiology of hypoxia‐related conditions such as long COVID, solid tumors, ischemia/ reperfusion injury, stroke, anaphylaxis, gastrointestinal disorders, and cardiovascular diseases [2, 3, 6, 7, 8]. In these settings, MCs have been implicated in shaping vascular permeability, leukocyte recruitment, and tissue remodeling, with outcomes that vary by organ and respective stimuli [5, 6, 7, 8, 9, 10, 11, 12, 13].

Despite this well‐established intersection, it remains unclear how resilient MCs are to hypoxia/ischemia and whether hypoxia–reoxygenation is sufficient to activate MCs independently of canonical FcεRI/Ag signaling [4, 14]. Prior reports have reached divergent conclusions regarding hypoxia‐induced degranulation [3, 9, 14, 15]. Addressing this gap is important because ischemia–reperfusion and other hypoxic inflammatory states are often dominated clinically by cytokine‐driven pathology, and MCs may play a strategic upstream role capable of amplifying or constraining these cascades. At the cellular level, adaptation to hypoxia is orchestrated primarily by hypoxia‐inducible factors (HIFs). Under normoxia, HIF‐1α is continuously synthesized but rapidly degraded, whereas under hypoxia, degradation is inhibited, enabling HIF‐1α stabilization, nuclear translocation, and transcriptional control of metabolic and pH‐adaptive programs, including induction of glucose transporters (e.g., GLUT1) and carbonic anhydrase IX (CAIX) [16, 17]. In MCs, HIF‐1α has been linked to activation in certain contexts, yet the timing, magnitude, and functional consequences of HIF signaling during hypoxia–reoxygenation—particularly in primary murine bone marrow–derived MCs — remain incompletely defined [6, 18]. Moreover, how canonical MC signaling checkpoints interface with hypoxic sensing pathways is largely unknown. SRC homology 2 (SH2) domain‐containing inositol 5′‐phosphatase 1 (SHIP1) is a key negative regulator of Ag‐mediated MC activation [10, 11]. SHIP1 regulates the intracellular levels of phosphatidylinositol‐3,4,5‐trisphosphate (PIP3), the product of phosphoinositide 3–kinase (PI3K), by PIP3 dephosphorylation at the 5'‐position, generating phosphatidylinositol‐3,4‐bisphosphate [12]. *Ship‐1*‐deficient (*Ship1^−/−^*) BMMCs are more prone to Ag‐mediated degranulation in comparison to wild‐type (*Ship1^+/+^*) BMMCs [13, 19, 20]. Ag‐triggered production of IL‐6 and TNF‐α has been described to be also enhanced in *Ship1^−/−^* BMMCs compared with *Ship1^+/+^* BMMCs [16, 17, 18, 19], supporting a role for SHIP1 as a “gatekeeper” of MC effector functions [13, 16]. These observations raise the possibility that SHIP1 may also function as a checkpoint in Ag‐independent MC activation programs, including those initiated by hypoxic stress and reoxygenation.

Here, we establish a controlled in vitro hypoxia–reoxygenation paradigm in primary mouse BMMCs to define (i) the intrinsic hypoxia tolerance of MCs relative to other primary murine cell types, (ii) whether hypoxia and reoxygenation engage MC cytokine secretion, and (iii) the role of SHIP1 in shaping these responses. Using *Ship1^+/+^* and *Ship1^−/−^* BMMCs, coupled to readouts of viability, degranulation, cytokine production/secretion, and HIF‐1α pathway engagement, we identify a hypoxia–reoxygenation–responsive cytokine program that is constrained by SHIP1. These findings position SHIP1‐regulated PI3K signaling as a mechanistic node linking hypoxic stress to MC cytokine output and suggest a framework for targeting MC‐driven inflammation in ischemia–reperfusion and other hypoxic inflammatory microenvironments.

## 2 Methods

### 2.1 Primary Mouse BMMCs From Ship1^+/+^ and Ship1^−/−^ Mice

Immature MC precursors were isolated from femoral bone marrow and differentiated in vitro into bone marrow‐derived mast cells (BMMCs) as previously described [21]. All experiments were performed using age‐ and sex‐matched littermates derived from heterozygous intercrosses (*Ship1* ^+/−^ × *Ship1^+/−^*). All mice had a mixed C57BL/6 × 129/Sv genetic background. Per standardized protocol [22] cells were cultured in RPMI 1640 medium with the addition of L‐glutamine (Thermo Fisher Scientific, #21875‐034) containing 1% X63Ag8‐653‐conditioned medium (source of IL‐3), 15% FCS (Capricorn, #FCS‐12A), 10 mM HEPES (SigmaAldrich, #H0887), 100 µM β‐mercaptoethanol (Sigma‐Aldrich, #M6250), 100 units/mL penicillin and 100 µg/mL streptomycin (Sigma‐Aldrich, #P0781). To ensure an enclosed differentiation, cells were analyzed via fluorescence‐activated cell sorting (FACS) for the co‐expression of FcεRI and CD117, using a FACS Canto II (Beckton Dickinson). A more than 95% FcεRI and CD117 double‐positive population was used for further experiments.

### 2.2 Peritoneal‐Derived Mast Cells

Primary murine MCs were isolated from the peritoneum using a well‐established protocol [21, 23]. Peritoneal‐derived mast cells (PMCs) were cultured in conventional cell culture medium RPMI1640 (Invitrogen, #21875‐0991) with 15% FCS (Capricorn, #FBS‐12A), 10 mM HEPES (pH 7.4), 50 units/mL penicillin and 50 mg/mL streptomycin, 100 µM β‐mercaptoethanol, 30 ng/mL IL‐3 from X63‐Ag8‐653 conditioned medium [24] and depending on its biological activity approximately 20 ng/mL stem cell factor from CHO culture supernatant [21].

All cell types were reseeded at 5 × 10^5^ cells/mL in fresh medium at weekly intervals.

### 2.3 Preparation of Primary Murine Neuroglial Cells and Fibroblasts

#### 2.3.1 Isolation of Primary Murine Neurons

The procurement of murine neurons was performed in collaboration with the University of Marburg according to established protocol [25]. Meninges‐free cortices of 13.5‐day old Swiss Webster mouse embryos (Janvier, Le Genest‐Saint‐Isle, France) were collected in Leibovitz L15 medium (PAA Laboratories, Pasching, Austria), homogenized, and pelleted. The pellet was resuspended in Neurobasal‐A medium (Life Technologies, Carlsbad, CA, USA) supplemented with B27 (Life Technologies), L‐glutamine (Lonza, Basel, Switzerland), and 100 U/mL of streptomycin and 100 U/mL of penicillin. The cell solution was filtered through a 70 µM nylon cell strainer (BD Biosciences, Bedford, MA, USA) and plated on polyethylenimine‐coated plates at a density of 9 × 10^5^ cells/mL. After 2 days in vitro, 1 µM arabinofuranosyl cytidine was added. Cells were used for experiments on days 7–8.

#### 2.3.2 Isolation of Primary Murine Embryonic Fibroblasts

13.5‐ to 14.5‐day‐old mouse embryos were procured and cultured in DMEM culture medium with 10% FBS, L‐glutamine, and 100 U/mL of streptomycin and 100 U/mL of penicillin according to an established protocol [26].

#### 2.3.3 Isolation of Primary Murine Microglia, Astrocytes, and Oligodendrocytes

The cerebral cortices were prepared from 1–3 day‐old rat pups as per previously established protocols to obtain microglia [27, 28], oligodendrocytes [27], and astrocytes [29, 30]. The tissues were dispersed in Dulbecco's phosphate‐buffered saline (PBS) containing 1% trypsin and 0.02% EDTA; Cell suspensions were filtered through a 50 µm nylon mesh and centrifuged at 400×*g* for 5 min. Cell pellets were re‐suspended in Dulbecco's modified Eagle's medium (DMEM) supplemented with 10% heat‐inactivated and charcoal‐stripped fetal calf serum (PAA, Austria), streptomycin (0.5%, Invitrogen, Germany), and fungizone (0.1%, Invitrogen). Cells were seeded in 75 cm^3^ cell culture flasks coated with poly‐L‐ornithine (Sigma, Munich, Germany) at a density of 1–2 × 10^6^ cells/cm^2^. Cultures remained for 3–4 days at 37°C in a humidified incubator with 5% CO_2_. Medium was changed every second day.

After 14 days in cell culture, cells were agitated at 100 rpm for 2 h at 37 C to remove debris, dead cells, and microglia. The supernatant was collected and pelleted at 1000 rpm for 5 min to yield microglia. The flasks containing the pellet were subsequently washed with PBS, incubated with 7 mL of growth medium for 1 h at 37°C and CO_2_, and placed on the shaker at 240 rpm and 37°C for 18 h to detach oligodendrocytes from the astrocyte monolayer. For obtaining oligodendrocytes, the collected medium was centrifuged for 10 min at 800 *g* to obtain a pellet and seeded at a density of 2 × 10^6^ cells⁄ well on poly‐L‐ornithine (Sigma)‐coated six‐well plates. After 1 h, cells were gently washed with PBS and incubated in DMEM with insulin: [5 lg⁄mL], transferrin: [5 lg⁄mL], sodium selenite: [25 ng/mL] (Boehringer, Ingelheim, Germany).

### 2.4 Hypoxic Stimulation

In the normoxia control group, we used RPMI with 5% FBS. One hour before the start of hypoxic stimulation, the cells were seeded into RPMI 1640 Medium (+)‐L‐glutamine (Roswell Park Memorial Institute RPMI, Thermo Fisher, Schwerte, Germany) without FBS; FBS was eliminated due to possible interference of growth factors from FBS with hypoxia‐induced cytokines. A concentration of 1.0 × 10^6^ viable cells in 1.5 mL per well was used for all experiments. Oxygen depletion was induced with inert N_2_ in a customized hypoxia chamber (Part#: C174, C21, Biospherix, Parish, NY, USA) as in our previous studies [31, 32]. During the experiment, the hypoxia chamber had ≤1% O_2_. To ensure stable hypoxia conditions, we monitored oxygen levels, temperature, and pressure within the chamber. An additional oxygen sensor was used to monitor the oxygen level in the media (#200001735, PreSens, Regensburg, Germany). The chamber itself was placed in a humidified incubator set to 37°C. Normoxia controls were kept in a separate incubator at 37°C, 5% CO_2_, and atmospheric pressure.

### 2.5 Cell Viability I

Cell viability was analyzed with previously established protocols [33]. After treatment, cells were incubated with Annexin V‐Alexa Fluor 647 (Alexis Biochemicals) in a culture medium for 20 min at 4°C in the dark. Immediately before analysis by flow cytometry, propidium iodide (1 µg/mL) was added, and the cells were analyzed on a flow cytometer (Canto II; BD Biosciences). This method was used to determine the viability of BMMCs, neurons, astrocytes, fibroblasts, and oligodendrocytes.

### 2.6 Cell Viability II

Cell Viability was quantified using the CellTiter‐Blue assay following the manufacturer's instructions (CellTiter‐Blue assay, Promega, Walldorf, Germany), following the predetermined hypoxia and reperfusion intervals. This assay relies on the cells’ capacity to convert a redox dye (resazurin) into a fluorescent compound (resorufin), which was measured at 590 nm using a microplate reader (Tecan GmbH, Männedorf, Switzerland). This method was used for viability analyses of *Ship1^+/+^* and *Ship1^−/−^* BMMCs.

### 2.7 Degranulation Assay (β‐Hexosaminidase Release)

To determine the degranulation rate, MCs were preloaded with 0.3 µg/mL IgE anti‐DNP overnight. After washing and resuspension in Tyrode's buffer adapted to 37°C for 30 min, cells were stimulated for 30 min at 37°C with the indicated concentrations of DNP‐HSA [200 ng/mL]. The degree of degranulation was determined by measuring the release of β‐hexosaminidase [34]. Degranulation assays cannot distinguish between degranulation‐mediated and cell death‐mediated release of granular enzymes; therefore, we first assessed cell viability. β‐hexosaminidase release cannot be quantitatively equated with cell death due to its enzymatic amplification; however, comparing the death rate and β‐hexosaminidase release provides a reasonable approximation.

### 2.8 Enzyme‐Linked Immunosorbent Assay

Enzyme‐linked immunosorbent assay (ELISA) was performed for the detection of total IL‐6 (BD Pharmingen, Heidelberg, Germany) and TNF‐α (R&D Systems, Wiesbaden‐Nordenstadt, Germany) according to the manufacturer's protocol. IL‐6 and TNF‐α were measured in supernatants. Levels of cytokines varied between experiments due to the genetic background or age of the cells.

### 2.9 Western Blot

Western blot (WB) analysis was performed as described in our previous work [32, 35]. After stimulation, cells were lysed in ice‐cold radioimmunoprecipitation assay buffer (RIPA) supplemented with phosphatase and proteinase inhibitors (complete, #1187358001, Roche, Basel, Switzerland). 30 µg of protein was loaded onto the SDS‐PAGE gel and separated. After transfer onto the PVDF membrane (Roche) and blocking with 5% skim milk (#T145.3, Roth, Karlsruhe, Germany) in Tris‐buffered saline containing 0.05% Tween‐20 (#8076.4, Roth), the membrane was incubated with the primary antibody in corresponding concentrations (Table 1). Following the incubation with the secondary horseradish‐conjugated antibody, we visualized the results using the ECL Plus Kit (Thermo Fisher Scientific, Waltham, MA, USA). β‐Actin served as a loading control. Densitometric analysis was performed using ImageJ (NIH, Bethesda, MD, USA).

**TABLE 1:** List of antibodies used for western blot and immunofluorescence.

Antibody against	Company	Order no.	Host	Dilution WB	Dilution IF
CAIX	Novusbio, Centennial, CO, United States	NB100‐417	Rabbit	1:1000	
CD117	Bioss Inc., Woburn, MA, USA	bs‐0672R	Rabbit		1:500
ERK	Cell Signaling, Danvers, MA, USA	#4696	Mouse	1:1000	
pERK1/2	Cell Signaling, Danvers, MA, USA	#4370	Rabbit	1:1000	
FcεRI	Thermo Fisher Scientific Inc., Waltham, MA, USA	14‐5898‐82	Armenian hamster	1:1000	1:500
GLUT1	Novusbio, Centennial, CO, USA	NB110‐ 39115	Rabbit	1:1000	
Hamster IgG	BIOZOL, Eching, Germany	LIN‐ZYH9251	Goat	1:1000	
HIF‐1α	Novusbio, Centennial, CO, USA	NB100‐479	Rabbit	1:1000	
pGSK3b	Cell Signaling, Danvers, MA, USA	#9336	Rabbit	1:1000	
p85	Cell Signaling, Danvers, MA, USA	#4292	Rabbit	1:1000	
β‐actin	Abcam, Cambridge, UK	ab3280	Mouse	1:1000	
Anti‐mouse IgG	GE Healthcare, Chicago, IL, USA	NXA931	Goat	1:4000	
Anti‐rabbit IgG	GE Healthcare, Chicago, IL, USA	NA934	Goat	1:5000	
Goat anti‐rabbit	Invitrogen	A11008	Goat	1:500	1:500
Goat anti‐mouse	Invitrogen	A11032	Goat	1:500	1:500
Donkey anti‐rabbit	Invitrogen	A21206	Donkey	1:500	1:500
Donkey anti‐goat	Invitrogen	A11058	Donkey	1:500	1:500

### 2.10 Immunofluorescence

Immunofluorescence (IF) analysis was performed according to the established protocol [32, 35]. Cells were seeded onto Poly‐L‐lysine‐covered coverslips. After stimulation, coverslips were fixed with 4% formaldehyde in PBS and permeabilized with 0.2% Triton X‐100. Subsequently, the slides were blocked with immunofluorescence blocking buffer (1% BSA, 2% FBS in PBS) and incubated with the primary antibody overnight at 4°C. The secondary antibody was incubated for 1 h at room temperature. Negative controls were incubated with only PBS buffer overnight. Cell nuclei were stained with 40,6‐Diamidin‐2‐phenylindol (DAPI) (Roth). Stainings were evaluated using a Leica fluorescence microscope (Leica, Wetzlar, Germany). A list of antibodies used for immunocytochemistry is given in Table 1. Picture analysis was performed using ImageJ. Fluorescence was compared based on integrated density (IntDen), which was calculated as: area of individual cell × mean fluorescence of cell. The graphs show the average IntDen of all cells per experiment. This method was used to compare fluorescence intensity independent of cell size.

### 2.11 Statistics

For data analysis and visualization, we used GraphPad Prism (Version 10.6, San Diego, CA, USA). The ROUT test was used to identify outliers. In the case of significances in normality and/or variance homogeneity, values were BOX‐COX‐transformed after the calculation of the optimal lambda and used for statistical analysis. Intergroup differences were tested by two‐ or three‐way ANOVA followed by Tukey post hoc test (multiple groups). Data are given as arithmetic means ± SD. *p* < 0.05 was considered statistically significant. The number of experimental and technical repeats is indicated in the corresponding figure legends, including the statistical tests employed.

### 2.12 Declaration of Generative AI and AI‐Assisted Technologies in the Manuscript Preparation Process

During the preparation of this work, the authors used OpenAI and Grammarly to improve language and readability. After using these tools, the authors carefully reviewed and edited the content as needed and take full responsibility for the content of the published article.

## 3 Results

### 3.1 Primary Mouse BMMCs Exhibit Markedly Higher Hypoxia Tolerance Than Other Murine Primary Cell Types

**FIGURE 1:**
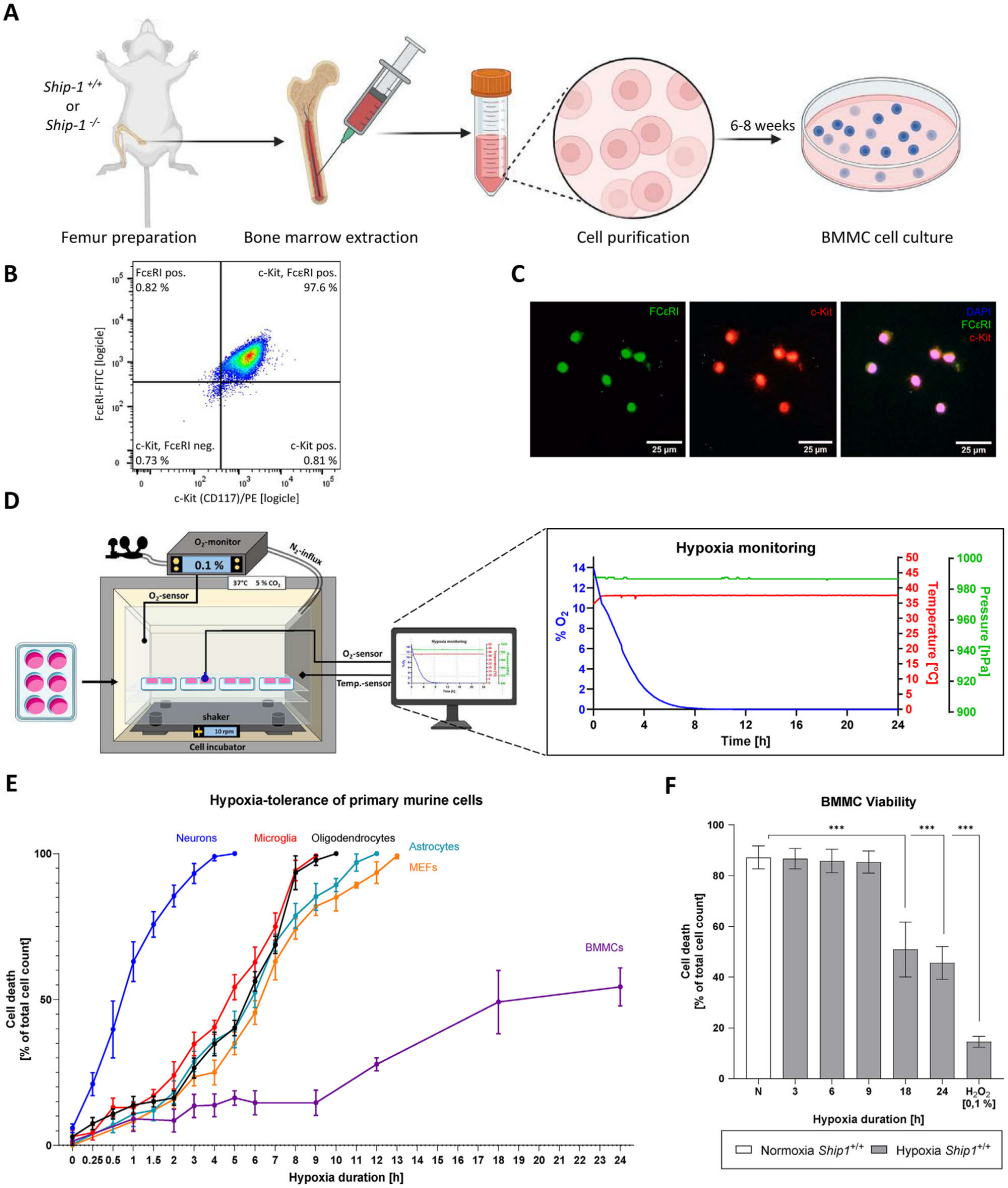
Primary mouse bone marrow mast cells (BMMCs) exhibit high hypoxia tolerance in comparison with other primary murine cells. (A) Schematic illustration of bone marrow‐derived mast cell (BMMC) generation. Characterization of BMMCs with flow cytometry and immunofluorescence for FcεRI (FITC‐A) and KIT (PE‐A) double positivity (B, C). Total number of events was at least 50,000 per FACS analysis. Magnification scale 20×. (D) Schematic illustration of customized hypoxia chamber and monitoring of environmental parameters including oxygen levels, temperature, pressure, humidity, and pH value in the media. Environmental parameters during BMMC and PMC hypoxia experiments (*n* = 3 independent experiments). (E) Hypoxia‐tolerance of primary murine neurons, microglia, oligodendrocytes, astrocytes, mouse embryonic fibroblasts (MEFs) and BMMCs. (F) BMMC viability measured via Annexin V FACS analysis after hypoxia and after stimulation with H_2_O_2_ [0.1%]. Intergroup differences were tested by two‐way ANOVA followed by a Tukey post hoc test (multiple comparisons). Bars represent means ± SD of three individual experiments with three technical replicates in each experiment; individual data points are shown. *p < 0.05, ^**^ *p* < 0.01, ^***^ *p* < 0.001 between groups, ^§^ *p* < 0.05 compared with shortest hypoxia or reoxygenation duration. Schematic presentation of mouse bone marrow–derived mast cell (BMMC) generation and validation by flow cytometry and immunofluorescence showing FcεRI and KIT double‐positive cells. Diagram of a controlled hypoxia chamber with monitored environmental parameters. Comparative plots show that BMMCs maintain higher viability under hypoxia than primary neurons, microglia, oligodendrocytes, astrocytes, and fibroblasts. Annexin V flow cytometry data indicate sustained BMMC viability after hypoxia with statistical comparisons across conditions.

To assess the impact of hypoxia on MCs, we first established a robust in vitro BMMC system and hypoxia–reoxygenation paradigm. BMMCs were differentiated in vitro and routinely confirmed to be mature by KIT and FcεRI co‐expression (≥97%) using flow cytometry and immunofluorescence (Figure 1A–C). To ensure reproducible hypoxic exposure, we used a custom hypoxia chamber with continuous monitoring of environmental parameters and direct sensing of oxygen and pH in the culture media (Figure 1D).

Across multiple primary murine cell types, BMMCs were substantially more resistant to oxygen deprivation than neurons, microglia, oligodendrocytes, astrocytes, and fibroblasts. Hypoxia‐induced cell death occurred fastest in neurons (complete by 3 h), followed by microglia and oligodendrocytes (∼50% lethality by 5 h) and astrocytes/MEFs (∼50% viable at 6 h). BMMCs were markedly more resistant, showing significant viability loss only after prolonged hypoxia (18 h) (Figure 1E,F).

**FIGURE 2:**
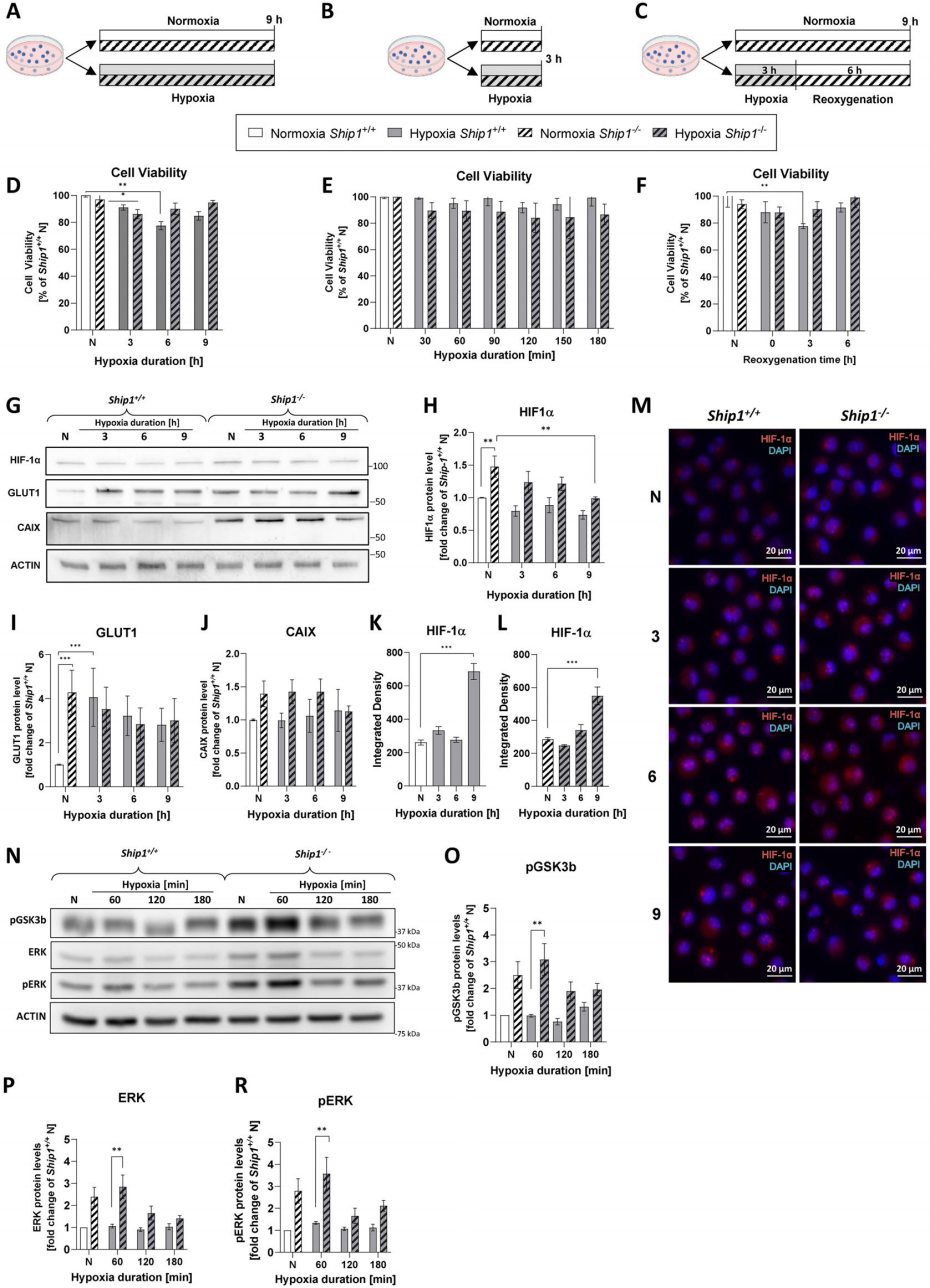
SHIP1 deficiency shifts the hypoxia‐response baseline and PI3K‐linked signaling readouts. Experimental workflow depicting the comparison of *Ship1* ^+/+^ vs *Ship1* ^−/−^ BMMCs in normoxia control group and (A) 9 h hypoxia, (B) short hypoxia intervals of up to 3 h, and (C) post‐3 h hypoxia reoxygenation. (D–F) BMMC viability measured via CellTiter‐Blue assay in corresponding experimental layouts. (G) Representative Western blot images of HIF‐1α, CAIX, GLUT1 and ACTIN protein levels in *Ship1* ^+/+^ and *Ship1* ^−/−^ BMMC after up to 9 h hypoxia. (H–J) Densitometric quantification of protein levels normalized to the normoxia control, where ACTIN was used as a loading control. (M) Representative immunofluorescence images of HIF‐1α (red) and DAPI in *Ship1* ^+/+^ and *Ship1* ^−/−^ BMMCs in up to 9 h hypoxia from three independent experiments. Magnification 40×. (K, L) Integrated density of HIF‐1α per >300 cells. (N) Representative Western blot images of pGSK3β, ERK, pERK, and ACTIN protein levels in *Ship1* ^+/+^ and *Ship1* ^−/−^ BMMC after 60, 120, and 180 min of hypoxia. (O–R) Densitometric quantification of protein levels normalized to the normoxia control, where ACTIN was used as a loading control. Intergroup differences were tested by two‐ or one‐way ANOVA followed by Tukey post hoc test (multiple comparisons). Bars represent means ± SD of three individual experiments; individual data points are shown. *p < 0.05, **p < 0.01, ***p < 0.001 between indicated groups. Workflow depicting the comparison of *Ship1^+/+^* vs *Ship1^−/−^* BMMCs in normoxia control group and 9 h hypoxia, short hypoxia intervals of up to 3 h, and post‐3 h hypoxia reoxygenation. Viability assays show differences between genotypes across conditions. Western blots and quantification demonstrate altered expression of hypoxia‐related proteins (HIF‐1α, CAIX, and GLUT1) and signaling proteins (pGSK3β, ERK, and pERK) in *Ship1^−/−^* BMMCs. Immunofluorescence images and quantification reveal increased HIF‐1α accumulation.

Having established the hypoxia resilience of BMMCs, we aimed to delineate the respective hypoxia‐response of BMMCs (Figure 2). It is known that MCs respond with cytokine release to various stress triggers (e.g., immunological, physical, chemical, infectious, and oxidative). Therefore, we aimed to determine whether solely hypoxia can induce cytokine secretion. SHIP1 acts as a degranulation gatekeeper by negatively regulating the PI3K pathway, which suggests its potential impact on MC activation beyond degranulation, as this pathway regulates multiple aspects of MC function including but not limited to cytokine production, survival, proliferation, adhesion, and migration. We next optimized hypoxia duration for mechanistic studies while minimizing cell death. In time‐course experiments, viability decreased earlier in *Ship1^−/−^* than in *Ship1^+/+^* BMMCs, prompting the selection of a 3 h hypoxia protocol for reoxygenation experiments to minimize cell type‐specific side effects (Figure 2A–F).

It is known that MCs exhibit the ability to express distinct receptors and tailor their responses based on the nature and intensity of the stimuli [36, 37]. Another frequently used model is PMCs, which are fully differentiated and tissue resident, in contrast to BMMCs, which are immature.

After cell isolation and cultivation, PMCs were characterized via FACS and immunocytochemically for c‐KIT (CD117) and FcεRI double positivity (Figure S4A–C). We determined that 3 h of hypoxia induced cytokine secretion without reducing viability in BMMCs; therefore, we subjected the cells to the now established experimental protocol with 3 h of hypoxia and subsequent 6 h reoxygenation, while normoxia served as the control group (Figure S4D). To delineate the MC response to hypoxia, we first established the viability of BMMCs and PMCs in experimental conditions (Figure S4E). We quantified the intracellular oxygen sensor HIF‐1α under hypoxic and reoxygenation conditions in both cell types and observed that BMMCs display higher posthypoxic activation of the Hif1α cascade than PMCs (Figure S4F–I).

To delineate the MC response to hypoxia, we first established the viability of BMMCs and PMCs in experimental conditions (Figure S4E). We quantified the intracellular oxygen sensor HIF‐1α under hypoxic and reoxygenation conditions in both cell types and observed that BMMCs display higher posthypoxic activation of the Hif1α cascade than PMC (Figure S4F–I).

### 3.2 SHIP1 Deficiency Shifts the Hypoxia‐Response Baseline and is Associated With Altered HIF‐1α‐Axis and PI3K‐Linked Signaling Readouts

Given the central role of HIF‐1α in hypoxia adaptation, we quantified HIF‐1α and canonical downstream markers (CAIX, GLUT1) under hypoxia and reoxygenation in *Ship1* ^+/+^ and *Ship1* ^−/−^ BMMCs. *Ship1* ^−/−^ BMMCs showed elevated baseline HIF‐1α protein levels by immunoblotting, with limited additional induction by hypoxia in that assay, whereas *Ship1* ^+/+^ BMMCs exhibited a hypoxia‐associated significant increase in GLUT1 (Figure 2G–J). We complemented immunoblotting with immunocytochemistry (ICC) to demonstrate the intranuclear translocation and stabilization of HIF‐1α and detected significant increases in HIF‐1α signal after prolonged hypoxia (e.g., 9 h) in both genotypes (Figure 2K–M; Figure S2). To link SHIP1 to signaling nodes upstream of HIF‐1α regulation, we examined PI3K/PIP3‐associated readouts. Phosphorylation of glycogen synthase kinase‐3β (pGSK3β) and extracellular regulated kinase (pERK) was enhanced, together with a significant increase in ERK protein levels after 60 min of hypoxia in *Ship1* ^−/−^ BMMCs compared with *Ship1* ^+/+^ BMMCs (Figure 2N–R). In summary, these findings demonstrate that *Ship1* ^−/−^ MCs show higher basal pERK and pGSK3β levels under normoxic conditions, which are further increased by hypoxia.

**FIGURE 3:**
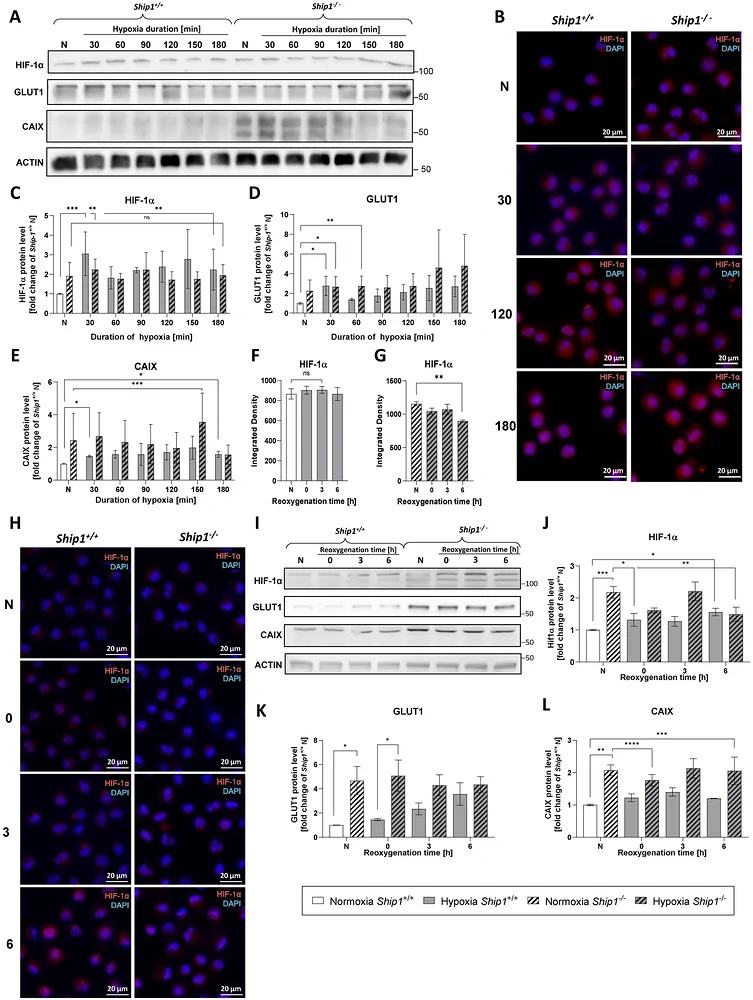
SHIP1 deficiency shifts alter the HIF‐1α‐axis in hypoxia/reoxygenation. (A) Representative Western blot images of HIF‐1α, CAIX, GLUT1 and ACTIN protein levels in short hypoxia intervals of up to 180 min. (B) Representative immunofluorescence images of HIF‐1α (red) and DAPI in *Ship1* ^+/+^ and *Ship1* ^−/−^ BMMCs in up to 180 min hypoxia from three independent experiments. Magnification 40×. (C–E) Densitometric quantification of protein levels normalized to the normoxia control, where ACTIN was used as a loading control. *Ship1* ^+/+^ (F) vs. *Ship1* ^−/−^(G) integrated density of HIF‐1α per >300 cells. (H) Representative immunofluorescence images of HIF‐1α (red) and DAPI in *Ship1* ^+/+^ and *Ship1* ^−/−^ BMMCs in post‐3 h hypoxia reoxygenation from three independent experiments. Magnification 40×. (I) Representative Western blot images of HIF‐1α, CAIX, GLUT1, and ACTIN protein levels in post‐3 h hypoxia reoxygenation. (J–L) Densitometric quantification of protein levels normalized to the normoxia control, where ACTIN was used as a loading control. Intergroup differences were tested by two‐ or one‐way ANOVA followed by Tukey post hoc test (multiple comparisons). Bars represent means ± SD of three individual experiments; individual data points are shown. *p < 0.05, **p < 0.01, ***p < 0.001 between indicated groups. Analysis of hypoxia and reoxygenation responses in *Ship1* ^+/+^ and *Ship1* ^−/−^ BMMCs over short hypoxia intervals (≤180 min) and after reoxygenation (post‐3 h hypoxia). Western blot and densitometry show genotype‐dependent differences in HIF‐1α, CAIX, and GLUT1 expression. Immunofluorescence images and quantification indicate altered HIF‐1α accumulation dynamics, especially in *Ship1* ^−/−^ cells during both hypoxia and reoxygenation.

HIF‐1α is constantly degraded and can rapidly be stabilized upon hypoxia [38, 39]. Therefore, we next investigated shorter hypoxia durations of 30–180 min to pinpoint the initial phase of this response. Western blot (WB) analysis showed a significant increase in HIF‐1α, GLUT1, and CAIX after 30 min of hypoxia in *Ship1* ^+/+^ BMMCs (Figure 3A,C–E). As mentioned before, *Ship1* ^−/−^ BMMCs displayed a higher baseline level of HIF‐1α, GLUT1, and CAIX compared with *Ship1* ^+/+^ BMMCs. ICC quantification showed a significant increase in HIF‐1α in *Ship1* ^−/−^ BMMCs after 30 min of hypoxia (Figure 3B; Figure S1A–D). These results suggest the involvement of SHIP1 in BMMCs and their response to hypoxia.

Having established that MC activation in hypoxia involves HIF‐1α stabilization, we sought to investigate HIF‐1α and its downstream CAIX and GLUT1 after reoxygenation. WB analysis showed an increase in HIF‐1α, GLUT1, and CAIX protein levels during reoxygenation in *Ship1^+/+^* BMMCs (Figure 3F−L). As described before, *Ship1^−/−^* BMMCs had higher HIF‐1α, GLUT1, and CAIX protein levels compared with *Ship1^+/+^* BMMCs, potentially masking the changes in HIF‐1α, GLUT1, and CAIX levels upon extended reoxygenation in these cells. These results were confirmed with HIF‐1α ICC analysis (Figure 3F–H; Figure S3).

Together, these data indicate that SHIP1 deficiency shifts the basal signaling and hypoxia‐response set point in BMMCs, reflected by elevated baseline HIF‐1α‐axis markers (HIF‐1α, GLUT1, CAIX) and enhanced phosphorylation of ERK and GSK3β under hypoxia. This pattern is consistent with SHIP1 acting as a negative regulator of stress‐response signaling interfacing with hypoxic adaptation pathways.

### 3.3 Hypoxia‐Induced Cytokine Secretion is Amplified by SHIP1 Deficiency

**FIGURE 4:**
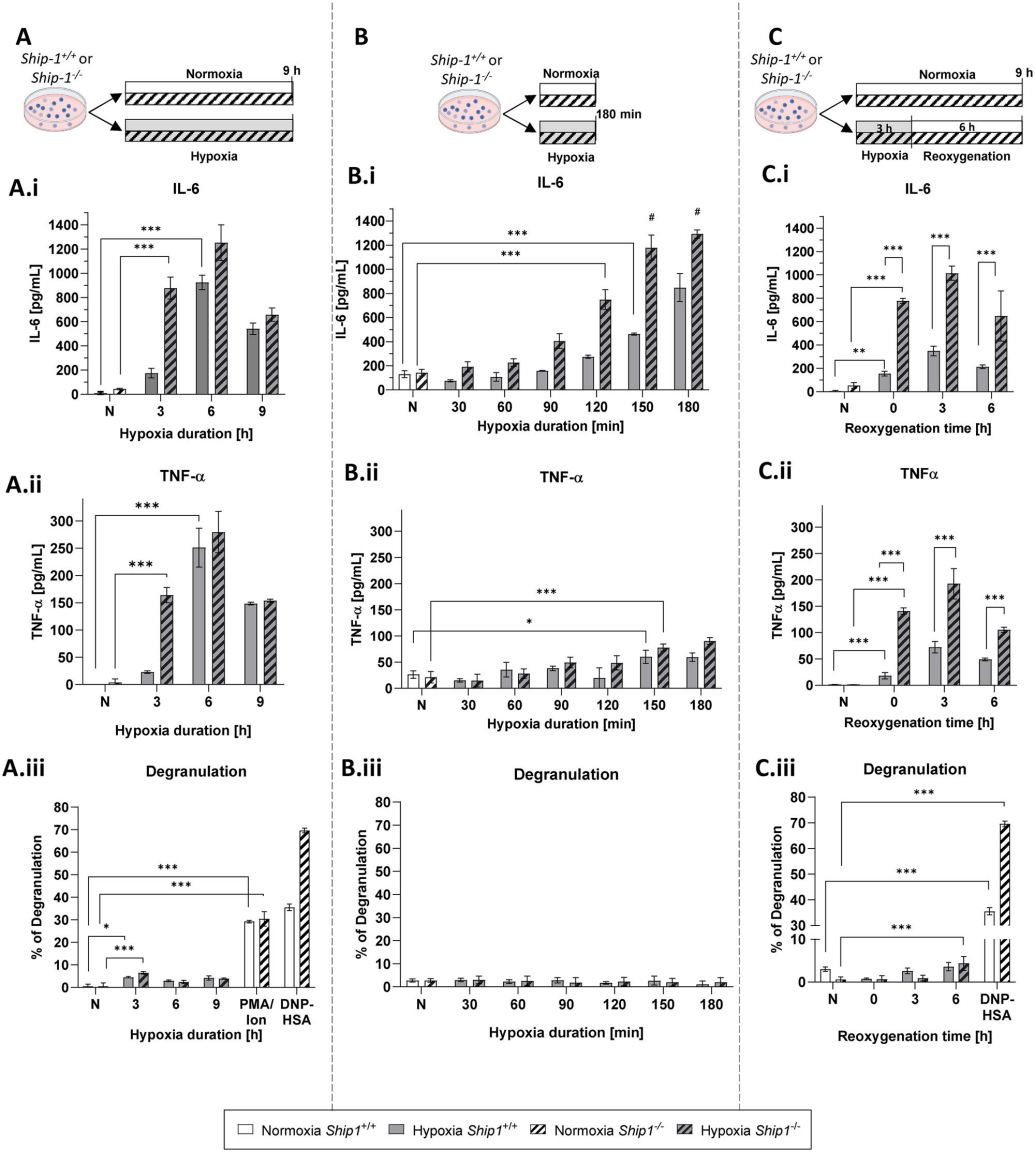
Hypoxia and reoxygenation induce amplified cytokine secretion in *Ship1* ^−/−^ BMMCs. Experimental workflow depicting the comparison of *Ship1* ^+/+^ vs. *Ship1* ^−/−^ BMMCs in the normoxia control group and (A) 9 h hypoxia, (B) short hypoxia intervals of up to 3 h, and (C) post‐3 h hypoxia reoxygenation. Posthypoxia (i) IL‐6 (pg/mL), (ii) TNF‐α concentrations (pg/mL), and (iii) degranulation rate (%) *Ship1* ^+/+^ vs. *Ship1* ^−/−^ BMMCs. Intergroup differences were tested by two‐ or one‐way ANOVA followed by Tukey post hoc test (multiple comparisons). Bars represent means ± SD of three individual experiments; individual data points are shown. *p < 0.05, **p < 0.01, ***p < 0.001 between indicated groups. Experimental workflow comparing *Ship1* ^+/+^ vs. *Ship1* ^−/−^ responses to 9 h hypoxia, short hypoxia intervals of up to 3 h, and post‐3 h hypoxia reoxygenation. Bar graphs show increased secretion of IL‐6 and TNF‐α, particularly in *Ship1* ^−/−^ BMMCs where degranulation is sustained.

Since BMMCs are known to engage two major response modes, cytokine secretion and degranulation, we next evaluated whether hypoxia is sufficient to induce either of these responses, and whether this is altered by SHIP1 deficiency. After 3 h of hypoxia, both *Ship1^+/+^* and *Ship1^−/−^* BMMCs showed increased secretion of IL‐6 and TNF‐α, and cytokine levels increased further at 6 h of hypoxia (Figure 4Ai, ii). Degranulation rates after hypoxia remained negligible (Figure 4Aiii). In contrast, as expected, in normoxia, PMA/ionomycin (200 ng/mL) induced about 30% degranulation in both genotypes, and the used antigen (DNP‐HSA [200 ng/mL], as expected [19], induced degranulation of ∼35% in *Ship1^+/+^* and ∼70% in *Ship1^−/−^* BMMCs (Figure 4Aiii).

To better pinpoint the onset of cytokine secretion during hypoxia, we performed a time course in 30 min increments up to 180 min. IL‐6 and TNF‐α levels increased progressively with longer hypoxia exposure and became significantly elevated after 150 min in both *Ship1^+/+^* and *Ship1^−/−^* BMMCs (Figure 4Bi, ii). Across these shorter intervals, no influence on degranulation was observed (Figure 4Biii).

Our data indicate that mere hypoxia is sufficient to induce IL‐6 and TNF‐α secretion in BMMCs, with a stronger cytokine response in *Ship1^−/−^* BMMCs.

### 3.4 Hypoxia‐Induced Cytokine Production/Release in BMMCs is Further Enhanced During the Reoxygenation Phase

To determine whether the response to hypoxia persists after restoration of oxygen, we analyzed IL‐6 and TNF‐α secretion and degranulation during the reoxygenation phase following hypoxic exposure. During reoxygenation, both *Ship1^+/+^* and *Ship1^−/−^* BMMCs showed enhanced cytokine secretion, with IL‐6 and TNF‐α peaking at 3 h of reoxygenation (Figure 4Ciii). Notably, *Ship1^−/−^* BMMCs secreted significantly more cytokines than *Ship1^+/+^* BMMCs at this peak time. In contrast to the cytokine response, *Ship1^+/+^* BMMCs did not show a significant increase in degranulation rates throughout reoxygenation for up to 6 h (Figure 4I). After 6 h of reoxygenation, *Ship1^−/−^* BMMCs displayed weak release of the granular enzyme β‐hexosaminidase compared with normoxia; whether this release is due to cell stress after prolonged hypoxia and subsequent reoxygenation remains to be analyzed. However, this increase remained substantially lower than degranulation induced by antigen (DNP‐HSA) (Figure 4Ciii).

Overall, these data indicate that reoxygenation following hypoxia maximizes IL‐6 and TNF‐α secretion (peak at 3 h) in both genotypes, with greater cytokine release in *Ship1^−/−^* BMMCs.

## 4 Discussion

Our study shows how mouse BMMCs respond to severe hypoxia (≤1% O_2_) and subsequent reoxygenation (21% O_2_), and identifies SHIP1 as a negative regulator of this response. We made three principal observations. First, BMMCs are remarkably resilient to oxygen deprivation compared with other primary murine cell types examined. Second, hypoxia triggers a robust inflammatory response characterized by IL‐6 and TNF‐α secretion without inducing degranulation under the same conditions. Third, this cytokine response is potentiated during reoxygenation and is consistently amplified in *Ship1* ^−/−^ BMMCs, together implicating SHIP1‐regulated signaling as a checkpoint that constrains posthypoxic MC activation.

A notable feature of our data is the high hypoxia tolerance of BMMCs relative to primary murine neurons, microglia, oligodendrocytes, astrocytes, and MEFs. This resilience likely reflects, at least in part, a rapid hypoxia adaptation program that includes reduced metabolic activity and engagement of hypoxia‐inducible factor (HIF) signaling [40]. In hypoxic tumor cells, HIF‐1 facilitates the shift of the glucose metabolism from oxidative phosphorylation to the glycolytic pathway (Warburg effect) [41]. More broadly, MCs are known to adapt their metabolic state and signaling repertoire to the needs of the local tissue microenvironment [31, 38], and may therefore be particularly well equipped to persist and function under oxygen‐limited conditions [36, 37]. In allergic reactions, IgE receptor cross‐linking activates intracellular signaling cascades, whereas in ischemia/reperfusion injury, MCs respond by complement activation and reactive oxygen species generation [3]. Conflicting reports exist on whether hypoxia induces MC degranulation; however, it has been postulated that the mechanism responsible for MC degranulation during ischemia/reperfusion injury differs from that triggered by allergens. During ischemia/reperfusion, the response is regulated by a series of G protein‐coupled receptors and substances produced by the MCs themselves, such as reactive oxygen species [42]. Our central functional finding is that hypoxia promoted cytokine secretion. This pattern aligns with prior work showing that hypoxia can support MC survival and mediator secretion with limited evidence for degranulation under comparable in vitro conditions [14]. By contrast, Steiner et al. [43] reported MC degranulation in vivo following systemic hypoxia (10% O_2_) with kinetics resembling compound 48/80 acting via MRGPRB2/X2. The discrepancy likely reflects the complexity of systemic hypoxia in vivo, where hypoxemia can trigger secondary mediators, vascular changes, and neurohumoral signals that are not present in isolated cell culture. In this regard, our controlled in vitro paradigm was designed to address whether oxygen deprivation per se is sufficient to induce MC activation. Within this experimental framework, hypoxia primarily engaged cytokine production and release rather than granule exocytosis.

We further expanded the study by comparing PMCs and BMMCs (Figure S4). They both showed comparable hypoxia tolerance in terms of viability, suggesting that both cell types behave similarly with respect to the main experimental time frame and support the overall validity of the model. In contrast, a distinct difference was observed regarding the HIF1α expression. This indicated that there could be cardinal differences in the response of both cell types to hypoxia, even though the tolerance is comparable. Further investigation is required into the comparison of tissue‐resident, mature PMCs and immature BMMCs.

Mechanistically, hypoxic sensing and adaptation are coordinated by HIF signaling. Under hypoxic conditions, rapid inhibition of HIF‐1α degradation enables its stabilization and transcriptional activity [38, 39]. HIF‐1α has been reported to increase following activation of calcineurin and NFAT signaling [44], while other work suggested that HIF‐1α stabilization in BMMCs may depend on hypoxia duration and experimental context [15]. Direct comparisons across studies remain difficult because stimuli, oxygen control strategies, and co‐incubation conditions vary substantially. Our approach therefore combined immunoblotting and immunocytochemistry to capture HIF‐1α–related dynamics across time scales. These methods provide the following perspectives: immunoblotting reports total lysate abundance, whereas ICC emphasizes stabilization and nuclear translocation through DAPI and HIF‐1α signal colocalization. At the same time, the detection of nuclear proteins, including HIF‐1α, by Western blot is highly influenced by the lysis conditions. All three experimental paradigms—hypoxia up to 180 min, prolonged hypoxia (9 h), and posthypoxia reoxygenation—contain a 3 h hypoxia time point. Although the direction of HIF‐1α induction was consistent, differences in signal intensity were observed for this shared time point and were reproducible across independent experiments. We attribute these discrepancies primarily to biological (cell variance due to primary cell cultures, cumulative cellular stress, rapid synthesis and proteasomal degradation, glucose availability) and technical (medium evaporation at long hypoxia exposure) differences in experimental designs. The regulation of HIF‐1α underlies several regulatory mechanisms, including instantaneous response with rapid degradation, metabolic adaptation, and negative regulation by oxygen‐sensitive prolyl hydroxylases [45].

CAIX and GLUT1 represent canonical effectors of the HIF‐1α cascade and provide insight into the metabolic and pH‐adaptive response. The pattern observed—early induction followed by attenuation under extended hypoxia, together with sustained elevation during reoxygenation—suggests a dynamic response shaped by oxygen availability and extracellular conditions. In particular, extracellular pH has been reported to directly influence CAIX expression [46], whereas GLUT1 is regulated through HIF‐1α–linked transcriptional programs. The coordinated behavior of CAIX and GLUT1 therefore highlights the interplay between oxygen sensing, metabolic adaptation, and pH homeostasis during transitions from hypoxia to reoxygenation.

A central aim of this study was to determine how a canonical MC regulator—SHIP1—shapes hypoxia responses. SHIP1 is a multifunctional lipid phosphatase that reduces intracellular PIP3 levels and thereby constrains PI3K‐dependent downstream signaling, influencing MC activation, survival, and degranulation [12, 13, 16]. SHIP1 has been characterized as a “gatekeeper” of MC degranulation and a negative regulator of antigen‐driven cytokine production [10, 11, 13, 16, 19, 20]. In line with this literature, SHIP1 deficiency in our system was associated with an amplified inflammatory phenotype, including increased IL‐6 and TNF‐α secretion following hypoxic treatment and during reoxygenation. In parallel, *Ship1* ^−/−^ BMMCs exhibited higher baseline levels of HIF‐1α pathway markers and increased phosphorylation of signaling intermediates including ERK and GSK3β under hypoxic conditions.

These findings support a model in which SHIP1 constrains hypoxia‐associated MC activation through signaling pathways that intersect with HIF‐1α regulation. PI3K signaling has been reported to upregulate HIF‐1α protein translation through AKT and mTOR‐dependent mechanisms [41], and ERK activation under hypoxia has been linked to HIF‐1 activation in other cell types [47]. Within this conceptual framework, the elevated baseline HIF‐1α, GLUT1, and CAIX levels in *Ship1* ^−/−^ BMMCs are consistent with altered PI3K‐proximal regulation, while the observed ERK and GSK3β phosphorylation patterns provide additional signaling correlates. Prior studies further support the concept of SHIP1 deficiency producing a hyperresponsive MC state with increased baseline cytokines and heightened responsiveness in vivo [48], consistent with our observation of augmented cytokine release in *Ship1* ^−/−^ BMMCs in response to hypoxia and reoxygenation. At the same time, our data indicate that SHIP1 prominently affects cytokine secretion, whereas release of granular β‐hexosaminidase remains negligible under hypoxia—presumably due to minimal cell death. Together, these findings delineate a SHIP1‐sensitive activation program that can be engaged by hypoxic stress.

Reoxygenation emerged as a critical amplifying phase in our system. Our findings are in agreement with previous work demonstrating that hypoxia modulates mast cell function. Segura‐Villalobos et al. [49] reported mast cell responses to cyclic hypoxia–reoxygenation in tissue and following IgE/antigen stimulation. Although their study employed a different experimental design, consisting of four cycles of 1 h at 1% O_2_ followed by 30 min reoxygenation in a tumor microenvironment, and subsequently assessed mast cell phenotype and reactivity following IgE/antigen stimulation, both studies consistently identify hypoxia as a key regulator of mast cell activation. While methodological differences exist compared with our in vitro approach, both studies consistently show hypoxia‐associated induction of HIF‐1α and IL‐6. While Segura‐Villalobos et al. [49] primarily analyzed transcriptional changes, we demonstrate corresponding alterations at the protein level.

Cytokine secretion peaked during reoxygenation, consistent with a sustained or potentiated activation state after hypoxia. Reoxygenation biology in MCs remains sparsely characterized [50]. One proposed mechanism is that HIF‐1α generated during oxygen‐glucose deprivation can become resistant to degradation after reoxygenation if prolyl hydroxylation is impaired by Krebs cycle metabolites such as fumarate and succinate [50]. In addition, conditioned media experiments have suggested that mediators released from hypoxia/reoxygenation‐treated MCs can exacerbate hypoxia/reoxygenation injury in intestinal epithelial cells via stress‐activated kinase pathways [51]. Within this broader context, our findings highlight reoxygenation as a period of intensified MC cytokine production and emphasize the need to consider hypoxia and reoxygenation as a coupled biological sequence rather than isolated states.

Several limitations should be acknowledged. As an in vitro system, our model does not capture key in vivo features of hypoxic disease niches, including dynamic oxygen gradients, low extracellular pH, reactive oxygen species, and elevated adenosine—factors that can modulate MC behavior in tumors and inflamed tissues [52]. Moreover, while we defined severe hypoxia as ≤1% O_2_ and reoxygenation as 21% O_2_, oxygen levels in solid tumors and ischemic tissues can vary across a broad range [53]. Future studies should extend these findings by testing graded oxygen tensions and chronic oxygen deprivation paradigms, and by incorporating in vivo models where MCs experience the full complexity of ischemia–reperfusion or tumor hypoxia. Moreover, different types of MCs should be analyzed.

Finally, these observations are relevant to disease contexts in which MCs have been implicated, and hypoxia is a core driver. In the CNS, meningeal MCs can worsen injury after stroke [6, 54], and recent work has highlighted MC‐linked signaling in poststroke inflammation via a dural–brain axis [55]. In other tissues, MC degranulation before ischemia has been described as protective in intestinal ischemia‐reperfusion [7], underscoring that the consequences of MC activation are context‐dependent. Therapeutic strategies that suppress MC activation have been proposed, including approaches targeting KIT, Siglec‐8, or pathways modulating activation states [56, 57]. Our data add SHIP1 as a candidate regulatory node in hypoxia–reoxygenation‐associated MC cytokine responses. While SHIP1/SHIP2 are being explored as therapeutic targets in cancer and hematologic malignancies [58, 59], their role as modulators of MC‐driven pathology in hypoxic inflammatory disease settings remains to be defined and warrants further investigation.

## Author Contributions

Conceptualization: Pardes Habib, Michael Huber. Data curation: Lara Gubeljak, Jonas Pes, Thomas Wilhelm, Aaron Fehr. Formal analysis: Lara Gubeljak, Jonas Pes. Funding acquisition: Pardes Habib. Investigation: Lara Gubeljak, Jonas Pes, Thomas Wilhelm. Methodology: Pardes Habib, Lara Gubeljak, Jonas Pes. Resources: Pardes Habib, Michael Huber, Thomas Wilhelm. Supervision: Pardes Habib, Michael Huber. Visualization: Lara Gubeljak. Writing – original draft: Lara Gubeljak. Writing – review and editing: Pardes Habib, Lara Gubeljak, Michael Huber, Thomas Wilhelm, Sandro Capellman, Jonas Pes, Eren Arik, Aaron Fehr, Mara Scheitinger. All authors contributed to the article and approved the submitted version.

## Funding

This work was funded by an internal grant (START grant 111/17, PH), the German Research Foundation (DFG), HA 9566/1–1 (PH), a grant by the Leonardis Stiftung (TW), and supported by the “Clinician Scientist program” of the Faculty of Medicine, RWTH Aachen University (PH). The funding body did not influence the design of the study, data acquisition, or analyses and interpretation of data.

## Ethics Statement

The procurement of primary murine cells followed German animal welfare regulations and the Principles of Laboratory Animal Care. Animals were maintained at the Institute of Laboratory Animal Science, Medical Faculty of RWTH Aachen University, which is licensed by the Veterinary Office of the Städteregion Aachen for laboratory animal husbandry and breeding and operates under a certified quality management system DIN ISO 9001/2015. All procedures involving mice were evaluated by the animal welfare officer and authorized by the Landesamt für Natur, Umwelt und Verbraucherschutz NRW (LANUV), Recklinghausen (AZ 84‐02.04.2016.A496). Mouse neurons were procured in collaboration with the University of Marburg, 35043 Marburg, Germany, in compliance with the local regulatory authority for animal regulations (LANUV, reference AZ‐2015.A095).

## Conflicts of Interest

The authors declare no conflicts of interest.

## Supporting information




**Supporting File 1**: eji70274‐sup‐0001‐SuppMat.pdf.

**Supporting File 2**: eji70274‐sup‐0002‐Figure‐captions.docx.

## Data Availability

The data that support the findings of this study are available from the corresponding author upon reasonable request.
