# Supplementary material for: SHIP1 Restrains Hypoxia/Reoxygenation‐Induced Cytokine Secretion in Mouse Bone Marrow–Derived Mast Cells
Source: Eur J Immunol. 2026 Sep 7;56(9):e70274. doi: 10.1002/eji.70274 (PMC13548442; doi:10.1002/eji.70274)
Supplement: Supplementary file 1 [file EJI-56-e70274-s001.pdf]

Supp. Fig. 1

A

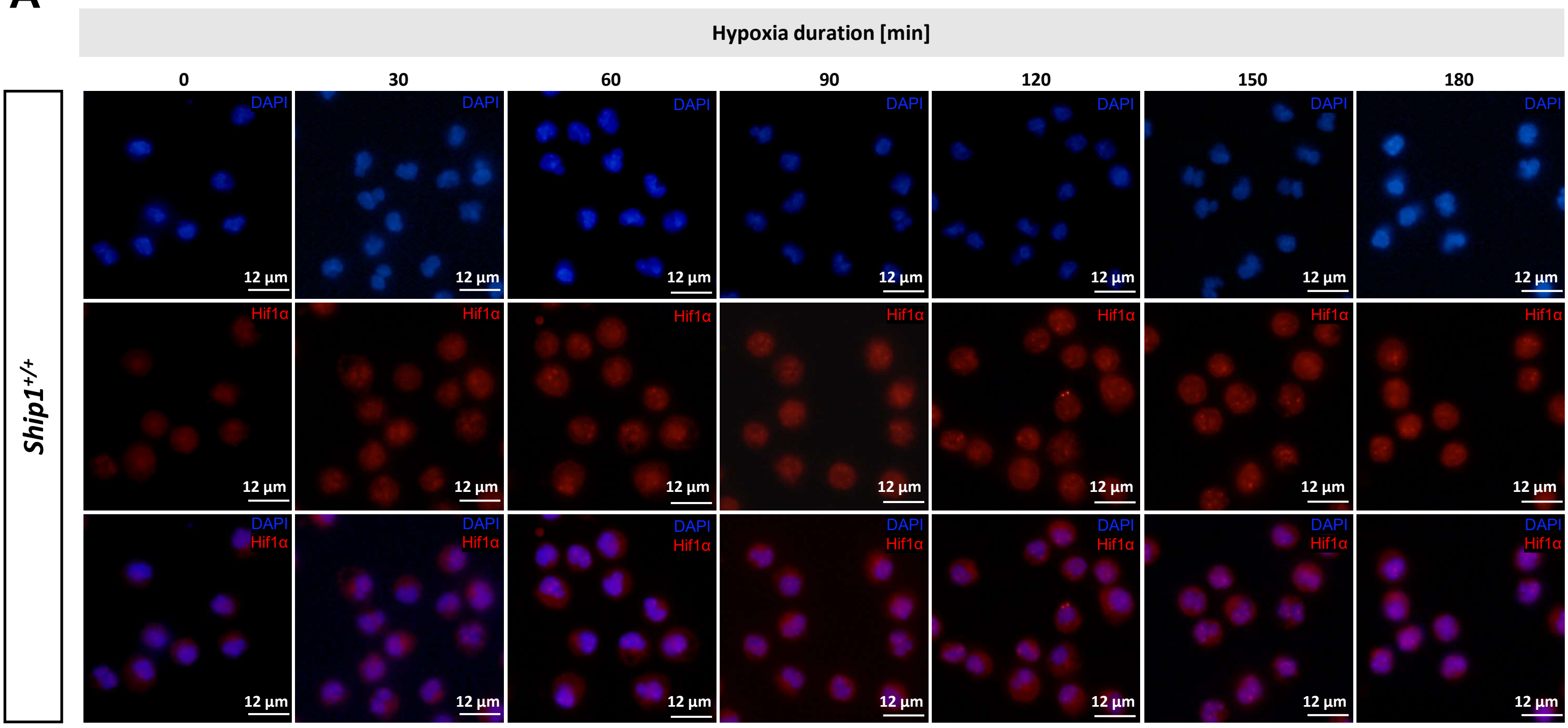

B

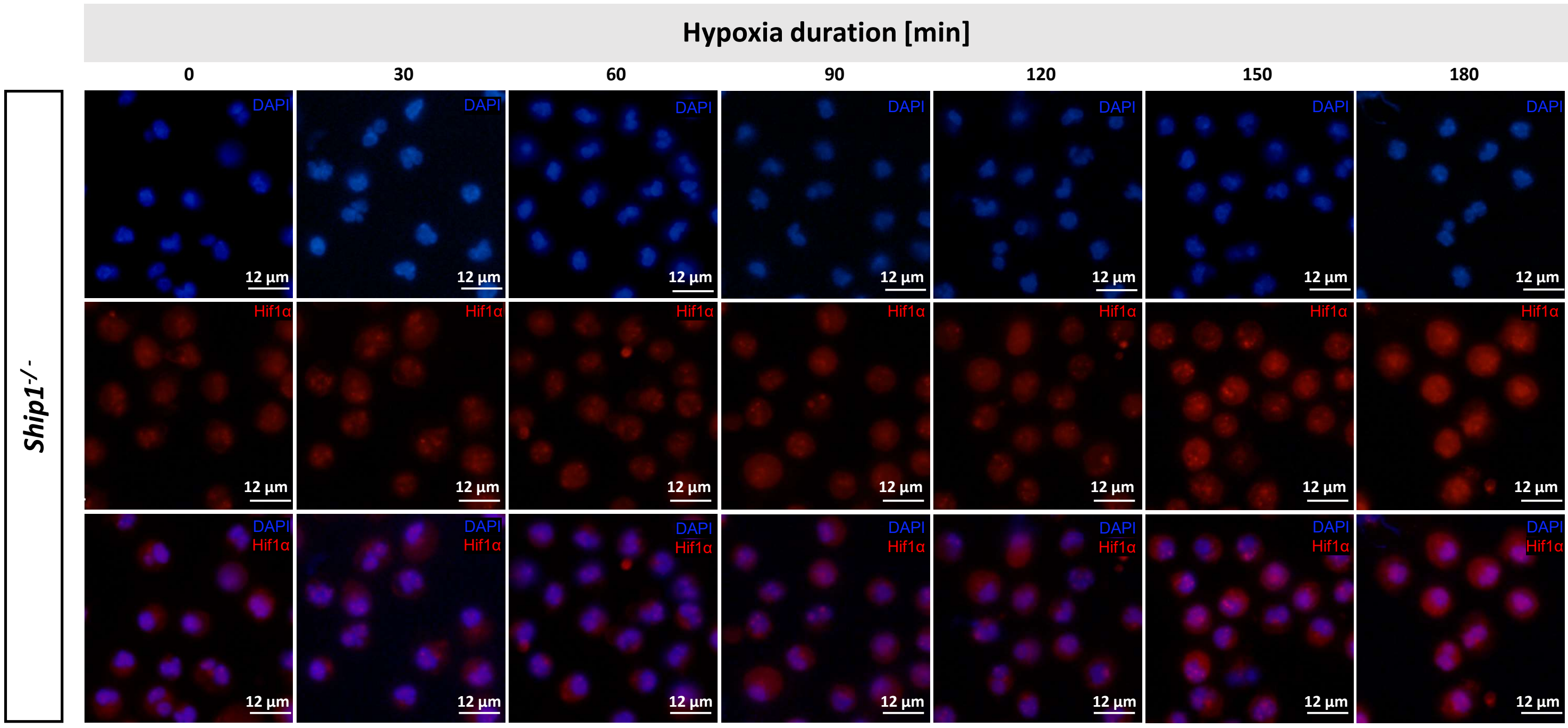

C

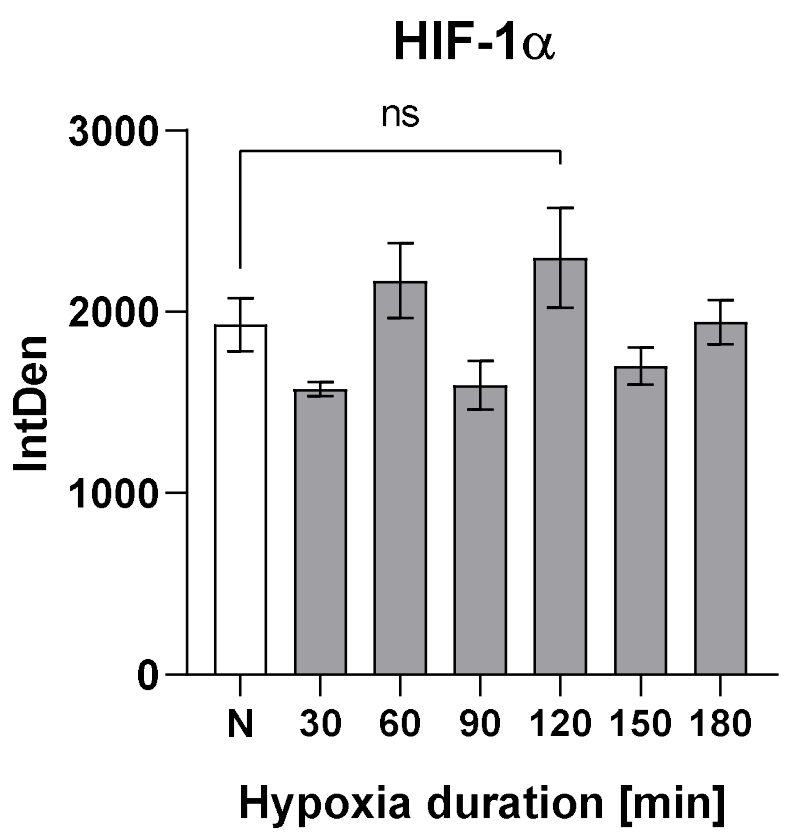

D

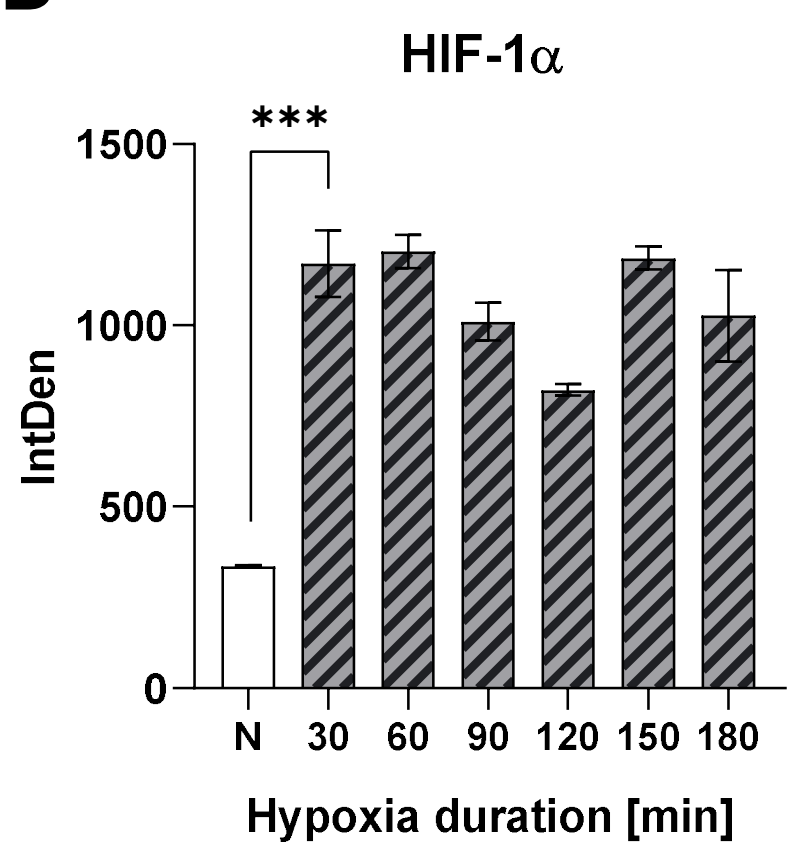

A

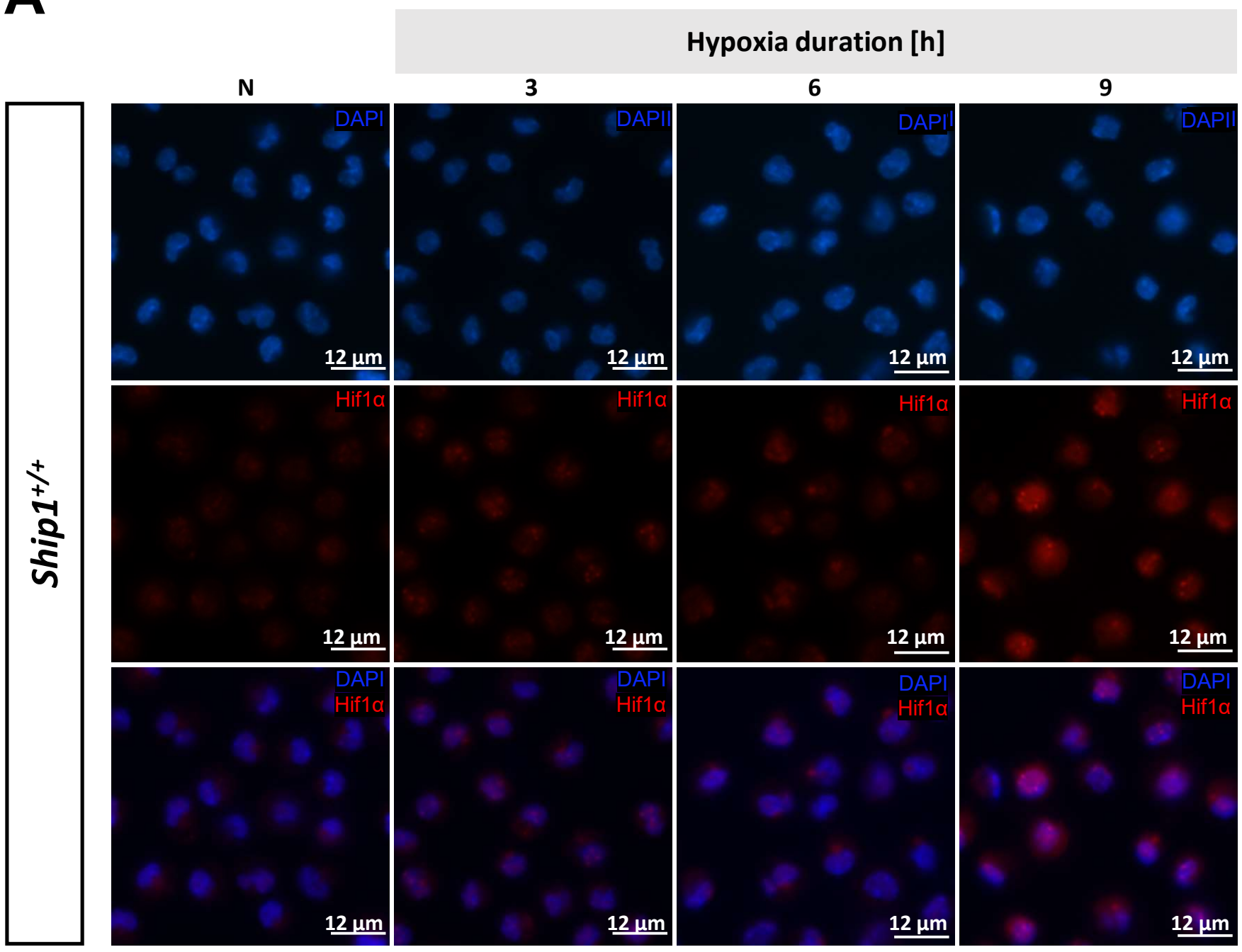
Hypoxia duration [h]
N
3
6
9
Ship1+/+
DAPI
Hif1α
12 µm

B

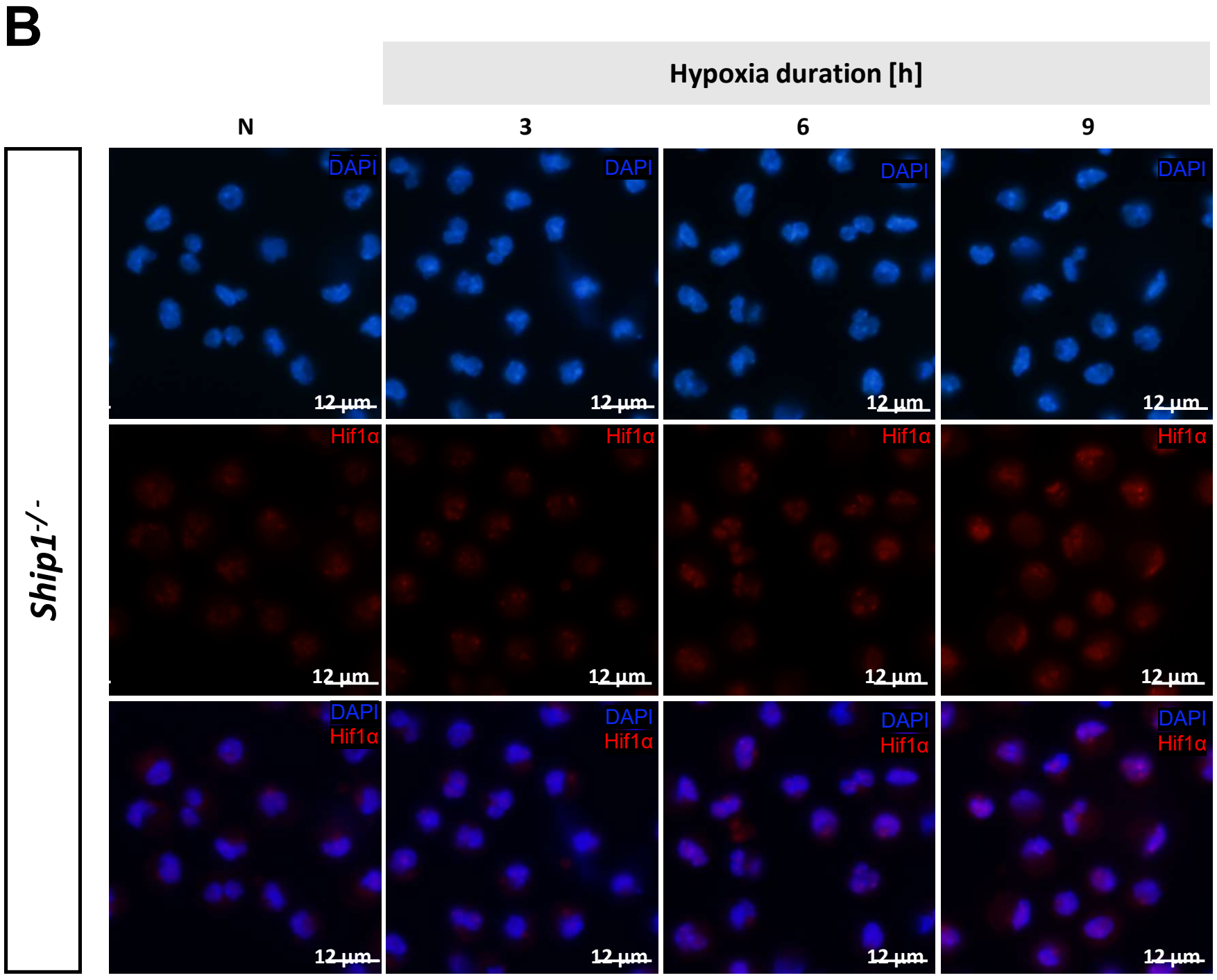
Hypoxia duration [h]
N
3
6
9
Ship1-/-
DAPI
Hif1α
12 µm

Supp. Fig. 3

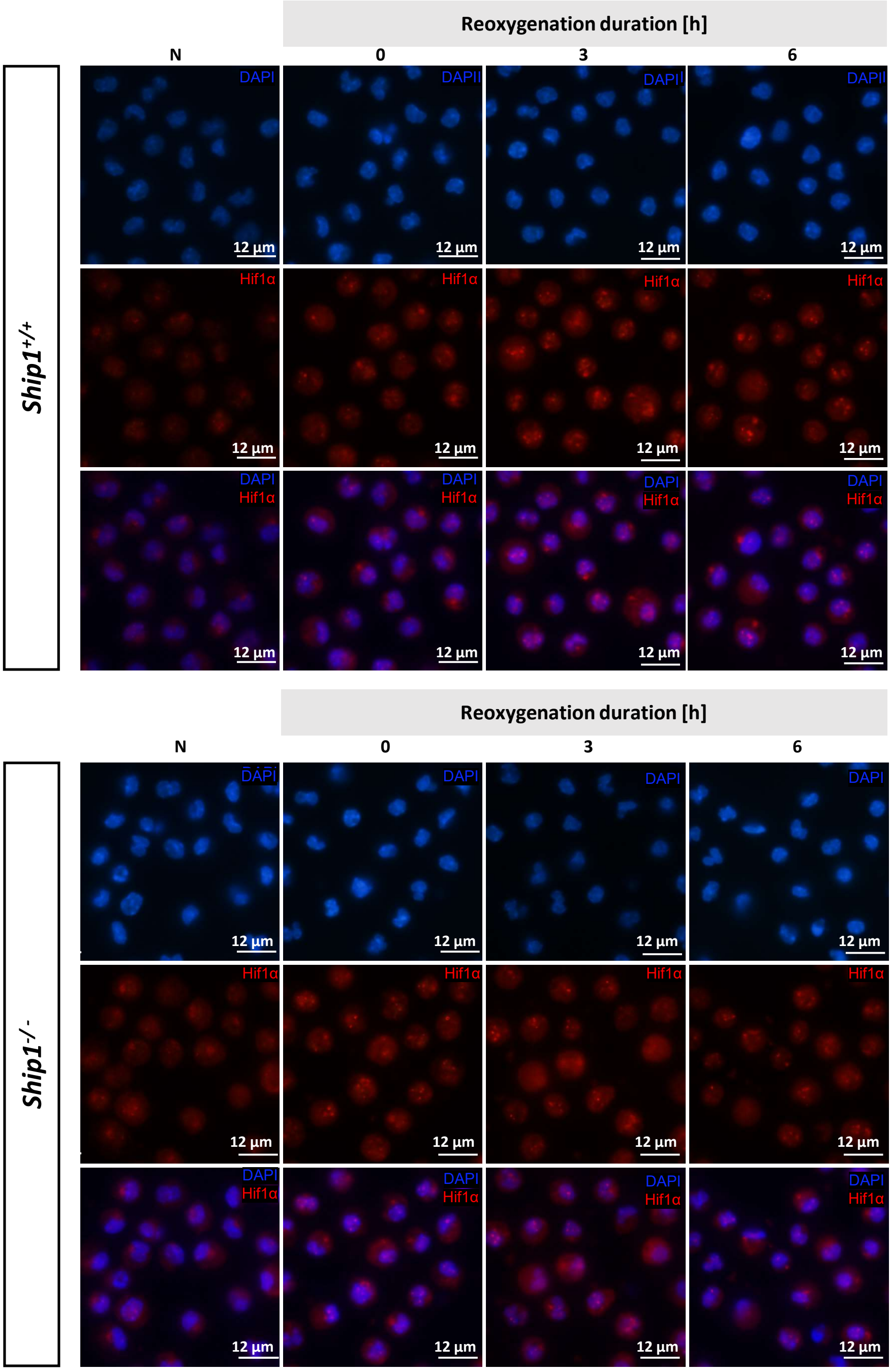

Reoxygenation duration [h]
N
0
3
6
Ship1+/+
DAPI
Hif1α
12 µm
Reoxygenation duration [h]
N
0
3
6
Ship1-/-

Supp. Fig. 4

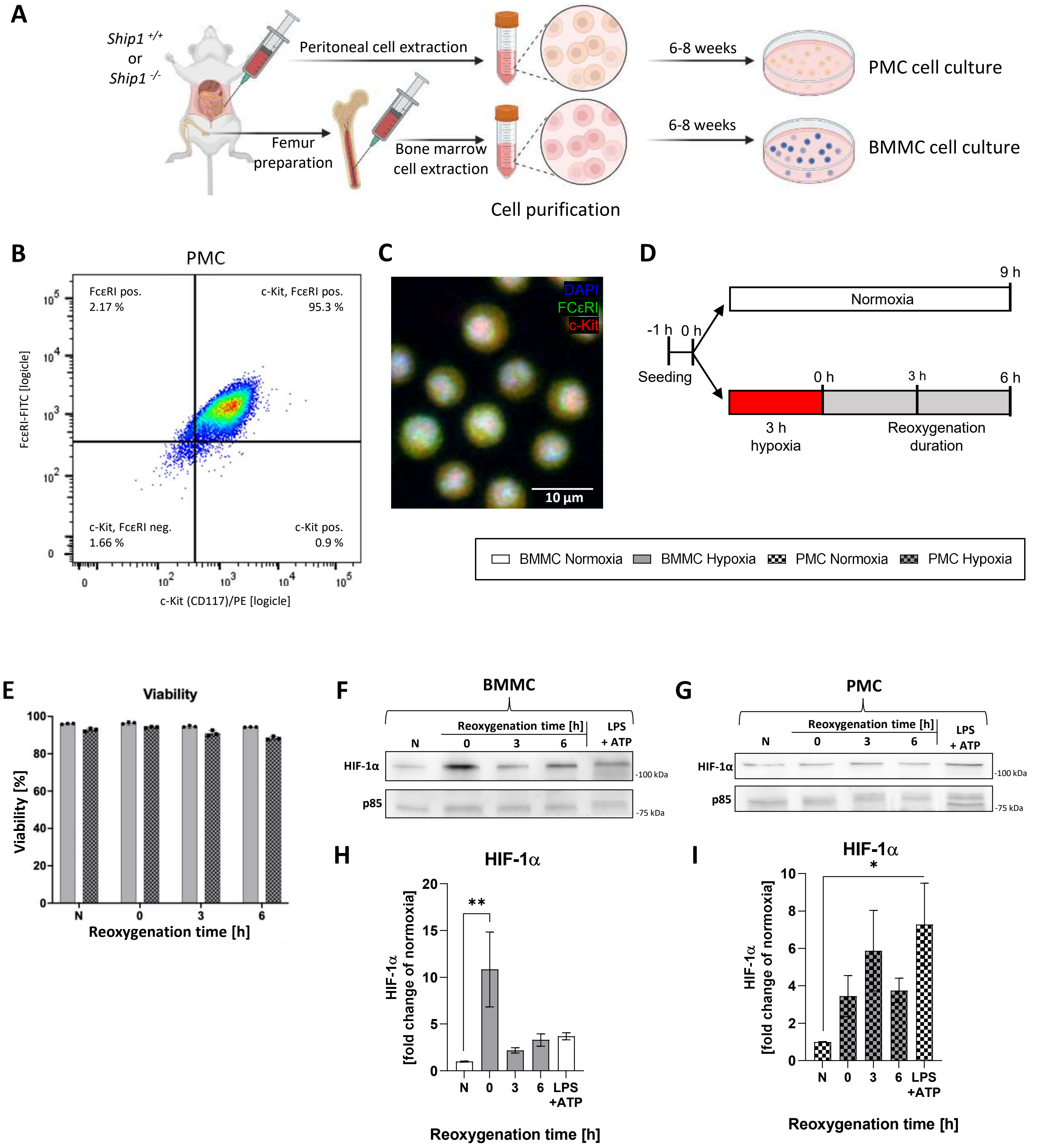
A
Ship1 +/+
or
Ship1 -/-
Peritoneal cell extraction
Femur
preparation
Bone marrow
cell extraction
Cell purification
6-8 weeks
PMC cell culture
6-8 weeks
BMMC cell culture
B
PMC
FcεRI pos.
2.17 %
c-Kit, FcεRI pos.
95.3 %
c-Kit, FcεRI neg.
1.66 %
c-Kit pos.
0.9 %
FcεRI-FITC [logicle]
c-Kit (CD117)/PE [logicle]
C
DAPI
FCεRI
c-Kit
10 µm
D
9 h
Normoxia
-1 h
0 h
Seeding
0 h
3 h
6 h
3 h
hypoxia
Reoxygenation
duration
BMMC Normoxia
BMMC Hypoxia
PMC Normoxia
PMC Hypoxia
E
Viability
Viability [%]
Reoxygenation time [h]
F
BMMC
Reoxygenation time [h]
LPS
+ ATP
HIF-1α
p85
-100 kDa
-75 kDa
G
PMC
Reoxygenation time [h]
LPS
+ ATP
HIF-1α
p85
-100 kDa
-75 kDa
H
HIF-1α
HIF-1α
[fold change of normoxia]
LPS
+ATP
Reoxygenation time [h]
I
HIF-1α
HIF-1α
[fold change of normoxia]
LPS
+ATP
Reoxygenation time [h]
