# Supplementary material for: SHIP1 Restrains Hypoxia/Reoxygenation‐Induced Cytokine Secretion in Mouse Bone Marrow–Derived Mast Cells
Source: Eur J Immunol. 2026 Sep 7;56(9):e70274. doi: 10.1002/eji.70274 (PMC13548442; doi:10.1002/eji.70274)
Supplement: Supplementary file 2 [file EJI-56-e70274-s002.docx]

**Supplementary Figure 1:** BMMC HIF-1α response in hypoxia durations of up to 180 min. Representative immunofluorescence images of HIF-1α (red) and DAPI in **(A)** *Ship1^+/+^* and **(B)** *Ship1^-/-^* BMMC from 3 independent experiments. Magnification 40x. **(C, D)** Integrated density of HIF-1α per min 300 cells. Intergroup differences were tested by ANOVA two-way or ANOVA one-way followed by Tukey *post hoc* test (multiple comparison). Bars represent means ± SD of 3 individual experiments, individual data points are shown. **p* < 0.05, ^**^*p* < 0.01, ^***^*p* < 0.001 between groups, ^#^*p* < 0.05 compared to previous reperfusion duration.

**Supplementary Figure 1 Alt text:** Immunofluorescence images depicting individual channels HIF-1α (red), DAPI (blue) and merged channels in *Ship1^+/+^* and *Ship1^-/-^* BMMCs in hypoxia durations of up to 180 min. Bar graphs show the integrated density quantification.

**Supplementary Figure 2:** BMMC HIF-1α response in hypoxia durations of up to 9 h. Representative immunofluorescence images of HIF-1α (red) and DAPI in **(A)** *Ship1^+/+^* and **(B)** *Ship1^-/-^* BMMCs from 3 independent experiments.

**Supplementary Figure 2 Alt Text:** Immunofluorescence images depicting individual channels HIF-1α (red), DAPI (blue) and merged channels in *Ship1^+/+^* and *Ship1^-/-^* BMMCs during prolonged hypoxia up to 9 h.

**Supplementary Figure 3:** BMMC HIF-1α response after 3 h of hypoxia followed by reoxygenation. Representative immunofluorescence images of HIF-1α (red) and DAPI in **(A)** *Ship1^+/+^* and **(B)** *Ship1^-/-^* BMMCs from 3 independent experiments.

**Supplementary Figure 3:** Immunofluorescence images depicting individual channels HIF-1α (red), DAPI (blue) and merged channels in *Ship1^+/+^* and *Ship1^-/-^* BMMCs response after 3 h of hypoxia followed by reoxygenation.

**Supplementary Figure 4: (A)** Schematic illustration of the isolation of Bone Marrow derived Mast Cells (BMMCs) and Peritoneal Mast Cells (PMCs). **(B)** Characterisation of PMCs with Flow Cytomety. **(C)** Exemplary immunofluorescence picture showing cells with double positivity for FcεRI and c-Kit (CD117). Magnification scale 40x. **(D)** Experimental workflow indicating normoxia control group and a 3 h hypoxia group with variable reoxygenation times. BMMCs and PMCs were subjected to 3 h of hypoxia followed by a reoxygenation period of up to 6 h. **(E)** BMMC and PMC viability after hypoxia and reoxygenation. **(F, G)** Representative Western blot images of HIF-1α and p85 protein levels in BMMCs and PMCs respectively. **(H,I)** Densitometric quantification of protein levels normalized to the normoxia control, where p85 was used as loading control. Intergroup differences were tested by ANOVA two-way or ANOVA one-way followed by Tukey *post hoc* test (multiple comparison). Bars represent means ± SD of 3 individual experiments, individual data points are shown. **p* < 0.05, ^**^*p* < 0.01, ^***^*p* < 0.001 between groups, ^#^*p* < 0.05 compared to previous reperfusion duration.

**Supplementary Figure 4 Alt text:** Isolation and characterization of bone marrow-derived and peritoneal mast cells, experimental hypoxia–reoxygenation workflow, cell viability assessment, representative HIF-1α Western blots, and corresponding densitometric analyses demonstrating hypoxia-induced protein expression changes during reoxygenation.
